# Chemokine receptor 7 targets the vascular endothelial growth factor via the AKT/ERK pathway to regulate angiogenesis in colon cancer

**DOI:** 10.1002/cam4.2426

**Published:** 2019-07-26

**Authors:** Xiang Li, Xuemei Wang, Zitao Li, Zhen Zhang, Yixia Zhang

**Affiliations:** ^1^ Department of Ultrasonic Diagnosis The First Affiliated Hospital, China Medical University Shenyang Liaoning PR China; ^2^ Department of Orthopedic Surgery Hongqi Hospital, Mudanjiang Medical University Mudanjiang Heilongjiang PR China

**Keywords:** AKT, angiogenesis, colon, CXCR7, ERK, vascular endothelial growth factor

## Abstract

**Background:**

Studies have shown that CXCR7 is expressed in many tumors. The aim of the present study was to investigate the function of CXCR7 in colon cancer. Although evidence indicates that CXCR7 promotes angiogenesis in colon cancer, the mechanism involved in this process remains unclear.

**Methods:**

The expression of CXCR7 in colon cancer was evaluated by quantitative reverse‐transcription polymerase chain reaction and western blotting. After transfection, cell proliferation, migration, and lumen formation were measured in vitro. Immunohistochemistry and western blotting were used to identify the functional target of CXCR7 in vivo and in vitro.

**Results:**

In this study, CXCR7 was differentially expressed in four colon cancer cell lines. The proliferation and migration experiments showed that overexpression of CXCR7 enhanced cell growth and migration. Moreover, the tube formation assays showed that co‐culture of colon cancer cells overexpressing CXCR7 with human umbilical vein endothelial cells significantly promoted tube formation in the latter cells. Conversely, the stable knockdown of CXCR7 significantly reduced this malignant activity. In addition, we found that CXCR7 activates the AKT and ERK pathways in colon cancer cells. The phosphorylation of AKT and ERK, as well as the expression of the vascular endothelial growth factor, can be inhibited using the LY294002 and U0126 inhibitors. Furthermore, the angiogenic ability of CXCR7‐induced colon cancer cells was eliminated.

**Conclusion:**

Expression of CXCR7 contributes to colon cancer growth and angiogenesis, by activating the AKT and ERK pathways. CXCR7 provides a potential therapeutic target against colon cancer.

## 1 INTRODUCTION

In a recent study, Freddie estimated that there will be 18.1 million new cases and 9.6 million cancer deaths worldwide in 2018.^1^ About 3 804 000 new cancer cases and 2 296 000 cancer deaths were reported in China.^2^ Colon cancer is a malignancy of the digestive system and the third most common type among malignant tumors. In China, the incidence and mortality rates of colon cancer have been increasing annually.^3^, ^4^ The incidence rate was 27.08/100,000 ( 30.55/100,000 in males, 23.43/100,000 in females). The mortality rate was 13.13/100,000 (14.84/100,000 in males, 11.34/100,000 in females).^2^ Despite treatment with surgery, chemotherapy, and endocrine therapy, a large number of patients continue to experience disease recurrence and metastasis.

Chemokine receptors are involved in the development and metastasis of various tumors.^5^ Chemokine receptor 7 (CXCR7)—formerly known as RDC1—is the second receptor for C‐X‐C motif chemokine 12 (CXCL12)/stromal cell‐derived factor 1 (SDF‐1), with a 10‐fold higher binding capacity than CXCR4.^6^ It has been reported that CXCR7 is highly expressed in colon cancer.^7^ More recently, numerous studies confirmed that CXCR7 is also expressed in other types of cancer (eg, pancreatic, thyroid, prostate, breast, esophageal, hepatocellular carcinoma, bladder), promoting tumor growth and metastasis.^8^, ^9^, ^10^, ^11^, ^12^, ^13^, ^14^ Neovascularization is an essential step for tumor growth, invasion, and metastasis.^15^ Therefore, anti‐angiogenesis is considered crucial in the treatment of cancer.

Notably, CXCR7 exhibits low levels of expression in normal mature vascular endothelial cells. In contrast, it is highly expressed in endothelial cells of neovascularized tumors.^16^ Zhu^17^found that CXCR7 is mainly expressed in the blood vessels of thyroid cancer tissues. Moreover, studies investigating tumor angiogenesis have revealed the expression of CXCR7 in breast and lung cancer.^5^ Therefore, CXCR7 may promote the development of cancer through the regulation of angiogenesis. Experimental studies indicate that CXCR7 regulates tumor growth and angiogenesis in colon cancer independently of SDF‐1. CXCR7 has shown increased expression in animals treated with anti‐SDF‐1 vs controls.^18^ Of note, Wang et al reported that overexpression and inhibition of CXCR7 influenced the concentration of interleukin 8 (IL‐8) and vascular endothelial growth factor (VEGF), and vascular sprout formation.^19^ In addition, Hao observed that the expression of CXCR7 may be involved in the formation of tumor blood vessels, and noted that CXCR7 alters the expression of VEGF and IL‐8. Their results also indicated that changes in the epithelial‐mesenchymal transition process of CXCR7 bladder cancer are caused by the activation of the AKT and ERK signaling pathways.^14^ It is likely that CXCR7 may also exert a significant effect in the progression of other malignant tumors. However, the molecular mechanisms through which CXCR7 is involved in tumor angiogenesis in colon cancer remain unclear. Therefore, in the present study, we investigated the role of CXCR7 in simultaneously activating the AKT and ERK signaling pathways to promote angiogenesis in colon cancer.

## 2 MATERIALS AND METHODS

### 2.1 Cell culture of tumor lines

The Caco‐2, SW480, HCT116, and RKO cell lines, as well as human umbilical vein endothelial cells (HUVECs) were purchased from the Cell Bank of the Chinese Academy of Sciences (Shanghai, China). All cells were cultured in a 37°C incubator with 5% CO_2_.

### 2.2 Transfection of CXCR7

The Caco‐2 and SW480 cell lines were transfected with small interfering RNA (siRNA) and short hairpin RNA (shRNA) against CXCR7 (RiboBio, Guangzhou, China), respectively (denoted as Caco‐2^siRNACXCR7^, Caco‐2^siRNANC^, SW480 ^shRNACXCR7^, and SW480^shRNANC^). The following complementary oligonucleotide encoding siRNA and shRNA were designed to knock down CXCR7: siRNA, 5′‐ GGAAGAUCAUCUUCUCCUATT‐3′ (sense) and 5′‐UAGGAGAAGAUGAUCUUCCGG‐3′ (antisense); shRNA, 5′‐ GATCCCCGAGCTCACGTGCAAAGTCATTCAAGAGATGACTTTGCACGTGAGCTCTTTTT‐3′ (sense) and 5′‐ AGCTAAAAAGAGCTCACGTGCAAAGTCATCTCTTGAATGACTTTGCACGTGAGCTCGGG‐3′ (antisense). These two colon cancer cell lines were also prepared for transfection with the pCMV6 Entry‐CXCR7 plasmid or pCMV6 Entry vector (RiboBio) (denoted as Caco‐2^CXCR7OE^, Caco‐2^CXCR7NC^, SW480^CXCR7OE^, and SW480^CXCR7NC^). The cells were transfected using Lipofectamine 3000 (Invitrogen, Grand Island, NY) according to the instructions provided by the manufacturer.

### 2.3 Cell counting kit‐8 (CCK‐8) analysis

HUVECs (100 µL) were dispensed in 96‐well plates (2 × 10^3^ cells/well) and incubated for 24 hours. The supernatant of each transfected group was collected and used for the CCK‐8 assay (Dojindo, Kumamoto, Japan). Subsequently, the supernatants of the transfected cells were added to the corresponding wells, according to the following ratios: 0%, 20%, 40%, 60%, 80%, and 100%. The absorbance was detected at 450 nm using a microplate reader (Thermo Fisher Scientific).

### 2.4 5‐Ethynyl‐20‐deoxyuridine (EdU) assay

HUVECs were dispensed in 24‐well plates (2 × 10^5^ cells/well) and cultured to a normal growth stage. Each transfected group of cells was dispensed in the upper chamber (4 × 10^5^ cells/well). After 24 hours, EdU was added to HUVECs and cultured at 37°C for 2 hours. Subsequently, the cells were stained using the Cell Light EdU DNA imaging Kit (RiboBio).

### 2.5 Cell migration

Transfected cells (4 × 10^5^ cells/well) were plated in the lower chamber with 500 µL of medium and incubated overnight. HUVECs (1 × 10^5^ cells/well) were plated in the upper chamber, which was filled with 200 µL of medium not containing fetal bovine serum (FBS). After 6 hours of incubation at 37°C, the migrant cells were photographed and counted using a microscope.

### 2.6 Tube formation assay

Matrigel (200 µL) (BD Biosciences) was plated in the lower chamber and incubated for 30 minutes at 37°C. HUVECs were seeded in 24‐well plates (2 × 10^5^ cells/well). Subsequently, transfected cells (4 × 10^5^ cells/well) were plated in the upper chamber, which was filled with 200 μL of medium containing FBS. Following incubation at 37°C for 6 hours, the tube formation was counted and photographed using a microscope.

### 2.7 Quantitative reverse‐transcription polymerase chain reaction (qRT‐PCR)

Total RNA was extracted from each group using TRIzol (Invitrogen) for 5 minutes. The mRNA expression was measured using a Prime Script RT Master Mix and SYBR Premix Ex Taq II kit (TaKaRa). The expression of glyceraldehyde 3‐phosphate dehydrogenase (GAPDH) was used as an internal control. Gene expression was detected a template of cDNA using the following oligonucleotide primers which were designed and produced by Sangon Biotech (Shanghai, China): CXCR7, 5′‐TCTGCATCTCTTCGACTACTCA‐3′ (Forward) and 5′‐GTAGAGCAGGACGCTTTTGTT‐3′ (Reverser); VEGF, 5′‐AAGGAGGAGGGCAGAATC‐3′ (Forward) and 5′‐CACACAGGATGGCTTGAAG‐3′ (Reverser); GAPDH, 5′‐GAAGGTGAAGGTCGGAGT‐3′ (Forward) and 5′‐GAAGATGGTGATGGGATTTC‐3′ (Reverser). Relative mRNA expressions were calculated using a Light Cycler 480 instrument (Roche).

### 2.8 Western blotting

The transfected cells were lysed in 100 μL of ice‐cold RIPA buffer (Beyotime, Shanghai, China) for 15 minutes and collected by scraping into individual Eppendorf tubes. The antibodies and their dilutions were as follows: CXCR7 (1:1000), VEGF (1:2000), ERK (1:10000), p‐ERK 1:5000, GAPDH (1:2000) (Abcam, Cambridge, UK), and AKT (1:1000), and p‐AKT Thr308 (1:1000) (Cell Signaling Technology, Danvers, Mass). The protein bands were detected using an Image Lab software and displayed using photographic film.

### 2.9 Enzyme‐linked immunosorbent assay (ELISA)

The culture supernatants were collected from each group after 24 hours. An ELISA kit (R&D Systems) was used to estimate the concentration of VEGF, measured at an OD_450_ wavelength using a microplate reader (Thermo Fisher Scientific).

### 2.10 Tumor model

Thirty female nude mice aged approximately 4‐5 weeks were purchased from Beijing Vital River Laboratory Animal Technology Co., Ltd.). The protocols and procedures used in this experiment were reviewed and approved by the ethics and approved by the Department of Laboratory Animals of China Medical University. Each nude mice received a subcutaneous injection of 0.2 mL of FBS mixed with approximately 1 × 10^7^ cells. When the tumor reached 1 cm^3^, the mice were sacrificed and the tumors were collected.

### 2.11 Histopathological analysis

Sections of tumors were prepared for immune histochemical analysis, embedded in paraffin, and cut into slices. After dewaxing, dehydrating, repairing, inactivating, and blocking, the tissue sections were incubated with CD34 (1:2500), CXCR7 (1:200) antibody, or VEGF (1:100) antibodies (Abcam), overnight at 4°C. After incubation with the secondary antibody, the sections were stained using diaminobenzidine. Cytoplasmic staining of vascular endothelial cells was considered positive.

### 2.12 Statistical analysis

All data are expressed as mean ± standard deviation. All statistical analyses were performed using the SPSS version 19.0 software (SPSS). Statistical differences between groups were calculated using one‐way analysis of variance. *P* < .05 denoted statistical significance.

## 3 RESULTS

### 3.1 Expression of CXCR7 in colon cancer cells

**Figure 1:**
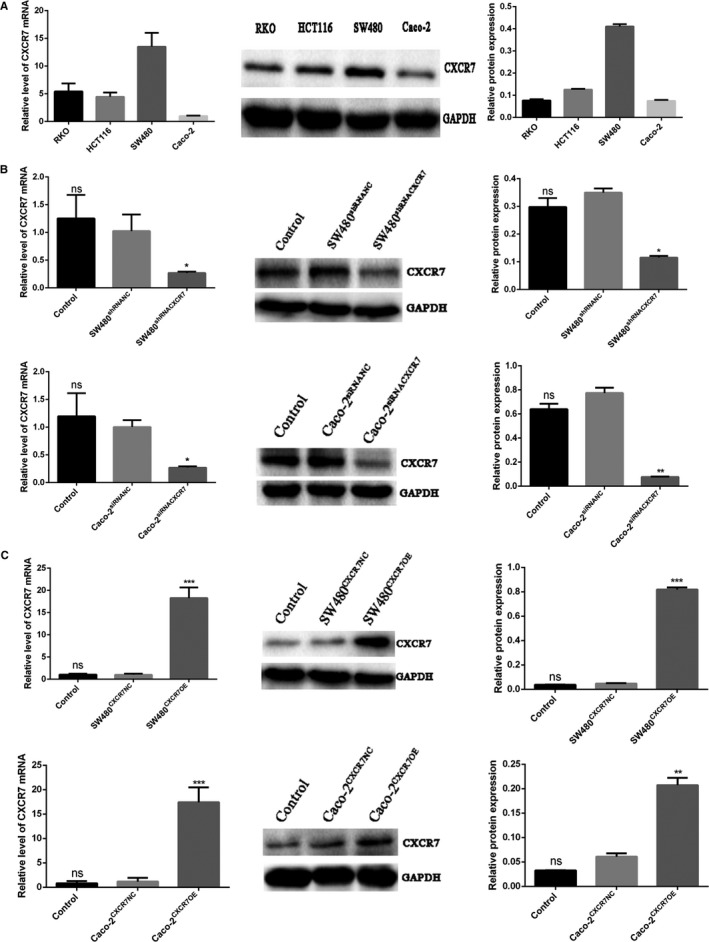
Expression of CXCR7 in colon cancer cells Notes: A, Expression of CXCR7 in RKO, HCT116, SW480, and Caco‐2 cells. B, Detection of decreased expression of CXCR7 in SW480 and Caco‐2 cells. C, Detection of increased expression of CXCR7 in SW480 and Caco‐2 cells. Data from each group are expressed as the mean ± standard deviation of three independent experiments. *P < .05, **P < .01, and ***P < .001 vs the respective NC group. Abbreviations: NC, negative control; ns, no statistical significance; CXCR7, chemokine receptor 7; CXCR7OE, overexpression of CXCR7

The expression of CXCR7 was evaluated in RKO, HCT116, SW480, and Caco‐2 cells using qRT‐PCR and western blotting. The results showed that CXCR7 was expressed in all four cell lines (Figure 1A). Notably, the expression of CXCR7 in SW480 and Caco‐2 cells was the highest and the lowest, respectively, among the four types of cells. SW480 and Caco‐2 cells were used for further analysis. CXCR7 was simultaneously overexpressed or silenced in SW480 and Caco‐2 cells. The protein and mRNA expression of CXCR7 was significantly lower in the silencing group (Figure 1B and Figure S1) and significantly higher in the overexpression group (Figure 1C and Figure S2) vs the negative control (NC) group. However, there was no significant difference in the expression of CXCR7 mRNA and protein between the control and the NC groups. These results indicate that CXCR7 is expressed in colon cancer.

### 3.2 Overexpression or silencing of CXCR7 affects tumor cell‐induced proliferation

**Figure 2:**
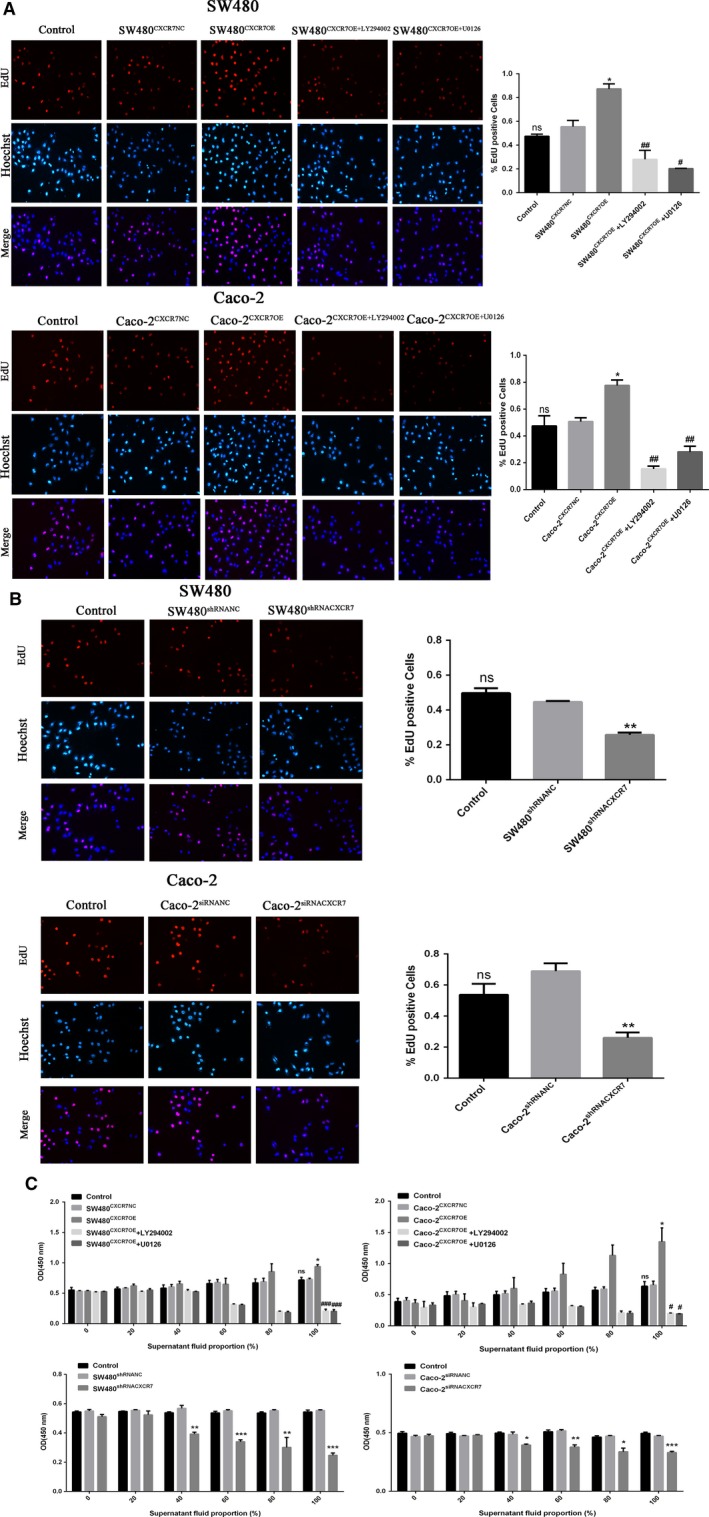
Effect of CXCR7‐mediated tumor cells on proliferation of HUVECs Notes: A, EdU assay showed that SW480 and Caco‐2 overexpressing CXCR7 promoted the proliferation of HUVECs, while inhibitors LY294002 and U0126 reduced their proliferation. B, EdU assay showed that SW480 and Caco‐2 silencing CXCR7 inhibited the proliferation of HUVECs. C, CCK‐8 was used to detect the proliferation of HUVECs in culture supernatants at different concentrations (0%, 20%, 40%, 60%, 80% and 100%). ^*^ *P* < .05, ^**^ *P* < .01 and ^***^ *P* < .001 vs each NC group. ^#^<.05, ^##^ *P* < .01 and ^###^ *P* < .001 vs overexpression group. Magnification 200×. Data in each group are expressed as mean ± SD from three independent experiments. Abbreviations: CCK‐8, Cell Counting Kit‐8; EdU, 5‐ethynyl‐20‐deoxyuridine; HUVECs: human umbilical vein endothelial cells; NC, negative control. ns: No statistical significance

To investigate the effect of CXCR7 on the growth of HUVECs in colon cancer, we examined their proliferative activity after co‐culture using EdU and CCK‐8 assays. The EdU assay showed a significant increase in the number of EdU‐positive HUVECs co‐cultured with SW480^CXCR7OE^ and Caco‐2^CXCR7OE^ vs the NC group (Figure 2A). In contrast, the number of EdU‐positive HUVECs co‐cultured with SW480^shRNACXCR7^ and Caco‐2^siRNACXCR7^ was significantly decreased compared with that observed in the NC group (Figure 2B). Furthermore, we did not observe a significant difference between the NC and control groups. The results of the CCK‐8 assay showed that the proliferation of HUVECs cultured in the supernatant of the overexpression group was significantly higher than that reported in the NC group. In contrast, the proliferation of HUVECs added to the supernatant of the silencing group was significantly reduced (Figure 2C). Moreover, the proliferation ability of HUVECs was enhanced in parallel with the concentration of cells in the supernatant. It was further found that LY294002 (a PI3K/AKT inhibitor) and U0126 (a MAPK/ERK inhibitor) downregulate the proliferation of CXCR7‐overexpressing cells. Collectively, our data indicate that changes in CXCR7 are significantly associated with the proliferation of HUVEC.

### 3.3 Overexpression or silencing of CXCR7 affects tumor cell‐induced migration

**Figure 3:**
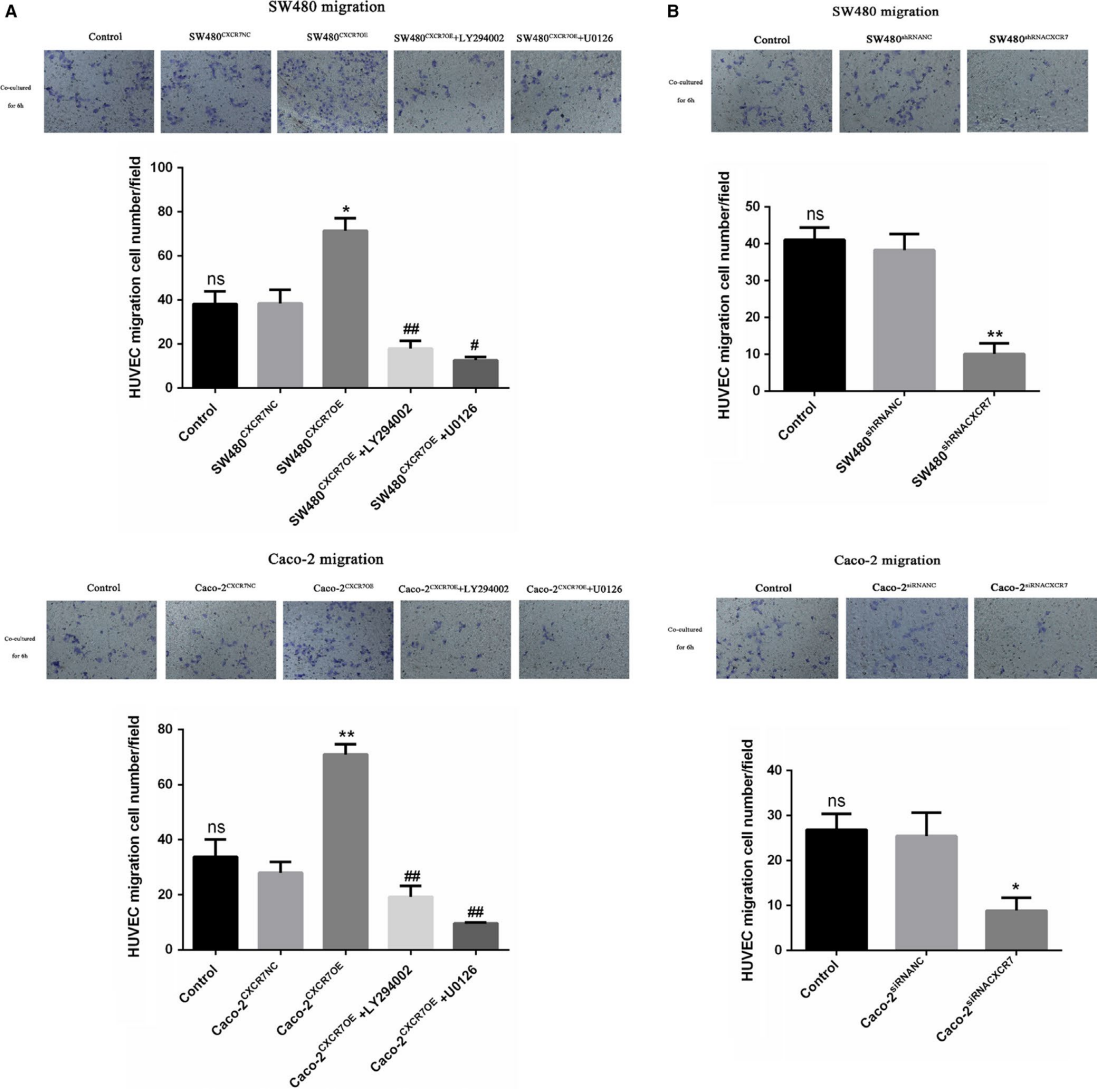
Effect of CXCR7‐mediated tumor cells on the migration of HUVECs Notes: A, Transwell migration results showed that SW480 and Caco‐2 cells overexpressing CXCR7 promoted the migration of HUVECs. Treatment with the inhibitors LY294002 and U0126 reduced the migration of HUVECs. B, Silencing of CXCR7 in SW480 and Caco‐2 cells inhibited the migration of HUVECs. ^*^ *P* < .05 and ^**^ *P* < .01 vs the respective NC group. ^#^ *P* < .05 and ^##^ *P* < .01 vs the respective overexpression group. Magnification 200×. Data in each group are presented as the mean ± standard deviation of three independent experiments. Abbreviations: HUVECs, human umbilical vein endothelial cells; NC, negative control; ns, no statistical significance; CXCR7, chemokine receptor 7; CXCR7OE, overexpression of CXCR7

Migration analysis was used to investigate the effect of CXCR7 expression on the migration of HUVECs in colon cancer. According to our results, the migration ability of HUVECs was significantly increased in the overexpression group (Figure 3A) and significantly decreased in the downregulation group (Figure 3B). In addition, there was no significant difference observed between the NC and control groups. Furthermore, it was found that the inhibitors LY294002 and U0126 downregulate the migration of CXCR7‐overexpressing cells. In conclusion, these results show that CXCR7 plays an important role in the migration of HUVEC.

### 3.4 CXCR7 overexpression or silencing affects tumor cell‐induced tube formation

**Figure 4:**
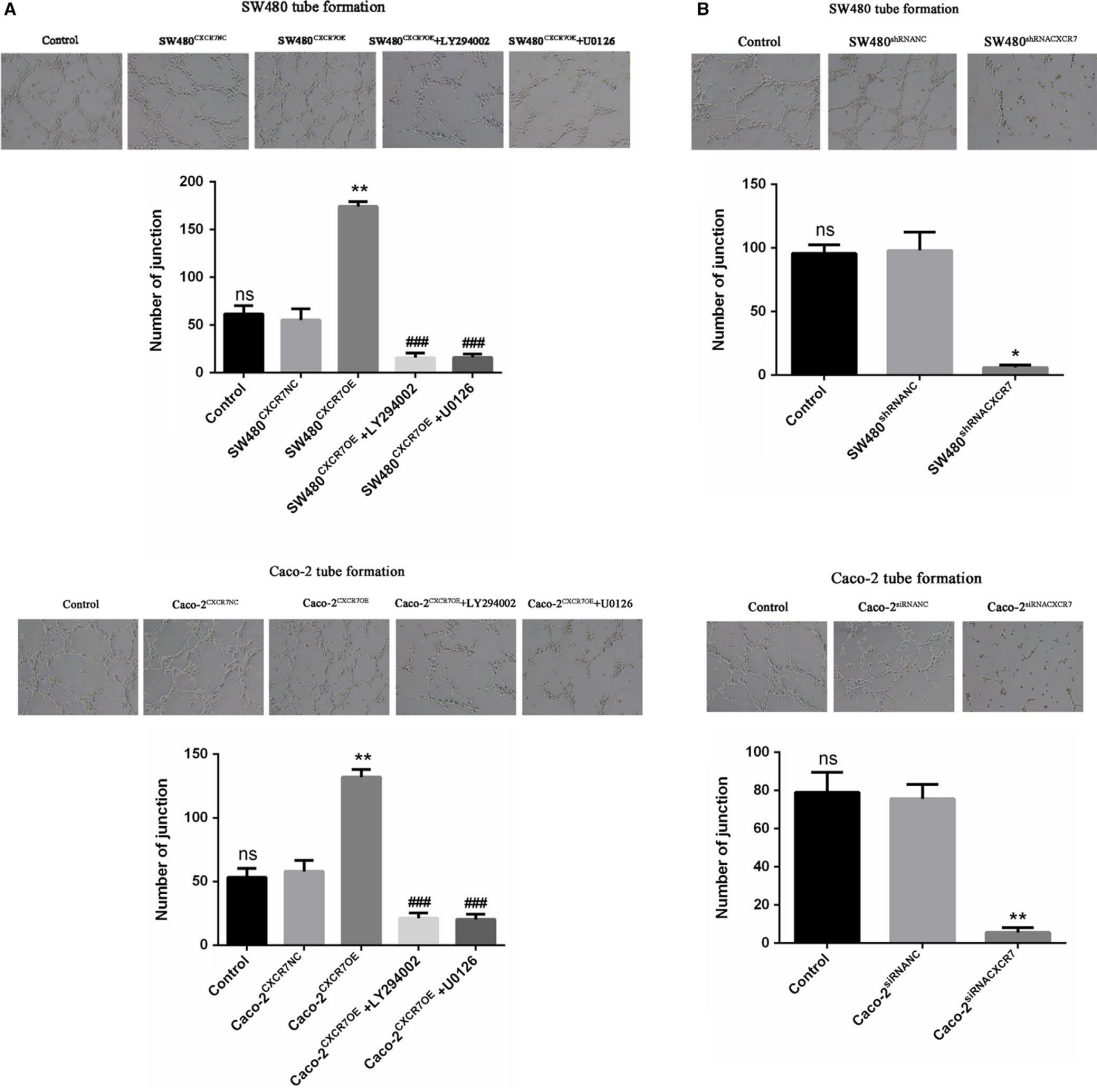
Effect of CXCR7‐mediated tumor cells on tube formation in HUVECs Notes: A, SW480 and Caco‐2 cells overexpressing CXCR7 promoted tube formation in HUVECs. Treatment with the inhibitors LY294002 and U0126 reduced tube formation in HUVECs. B, Silencing of CXCR7 in SW480 and Caco‐2 cells inhibited tube formation in HUVECs. ^*^ *P* < .05 and ^**^ *P* < .01 vs the respective NC group. ^###^ *P* < .001 vs the respective overexpression group. Magnification 200×. Data from each group are presented as the mean ± standard deviation of three independent experiments. Abbreviations: HUVECs, human umbilical vein endothelial cells; NC, negative control; ns, no statistical significance; CXCR7, chemokine receptor 7; CXCR7OE, overexpression of CXCR7

To explore the CXCR7‐induced tube formation in colon cancer, we quantified the tube formation in HUVECs on Matrigel by measuring the number of junctions. After co‐culture with the overexpression group, the angiogenesis ability of HUVECs was significantly enhanced. The number of junctions was significantly increased t that observed in the NC group (Figure 4A). In addition, HUVEC angiogenesis was significantly reduced in the silencing group (Figure 4B). Notably, the results between the control and NC groups were not statistically significant. Furthermore, it was found that the inhibitors LY294002 and U0126 downregulate the lumen formation ability of CXCR7‐overexpressing cells. The above data show that changes in CXCR7 in colon cancer are key factors for tube formation.

### 3.5 CXCR7 overexpression or silencing affects the secretion of VEGF

**Figure 5:**
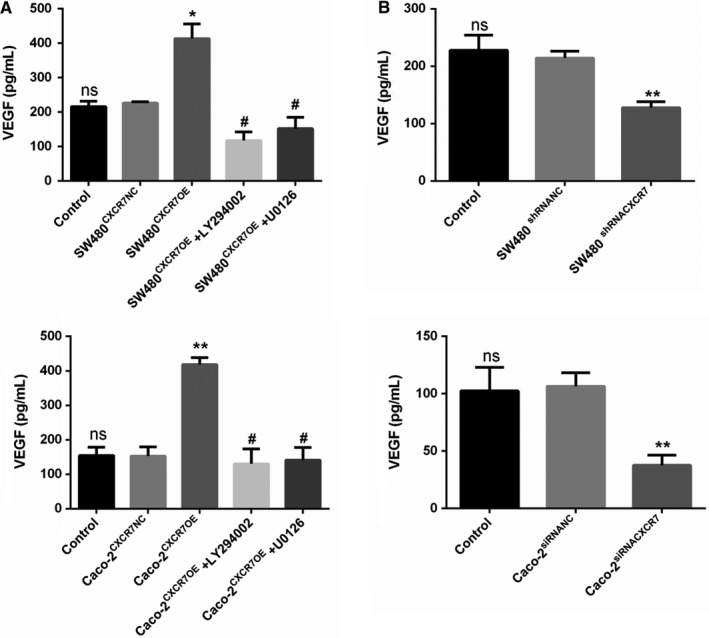
ELISA: Expression of CXCR7 regulates VEGF secretion Notes: A, The levels of VEGF were significantly increased in SW480 and Caco‐2 cells overexpressing CXCR7. Treatment with inhibitors LY294002 and U0126 reduced the levels of VEGF. B, The levels of VEGF in CXCR7‐silencing SW480 and Caco‐2 cells were significantly reduced. ^*^ *P* < .05 and ^**^ *P* < .01 vs the respective NC group. ^#^ *P* < .05 vs the respective overexpression group. Data from each group are presented as the mean ± standard deviation of three independent experiments. Abbreviations: ELISA, enzyme‐linked immunosorbent assay; VEGF, vascular endothelial growth factor; NC, negative control; ns, no statistical significance; CXCR7, chemokine receptor 7; CXCR7OE, overexpression of CXCR7

To further investigate the regulatory role of CXCR7 on pro‐angiogenic factors, we examined the concentration of VEGF in the supernatant fluid of various CXCR7 transfection groups through ELISA. The results showed that the concentrations of VEGF in CXCR7‐overexpressing SW480 and Caco‐2 cells were higher than those observed in the NC group (Figure 5A). In contrast, the levels of VEGF were significantly reduced in CXCR7‐silenced SW480 and Caco‐2 cells compared with those reported in the NC group (Figure 5B). Furthermore, it was found that the inhibitors LY294002 and U0126 downregulated the concentration of VEGF in CXCR7‐overexpressing cells.

### 3.6 Effects of CXCR7 expression on tumor growth and angiogenesis in vivo

**Figure 6:**
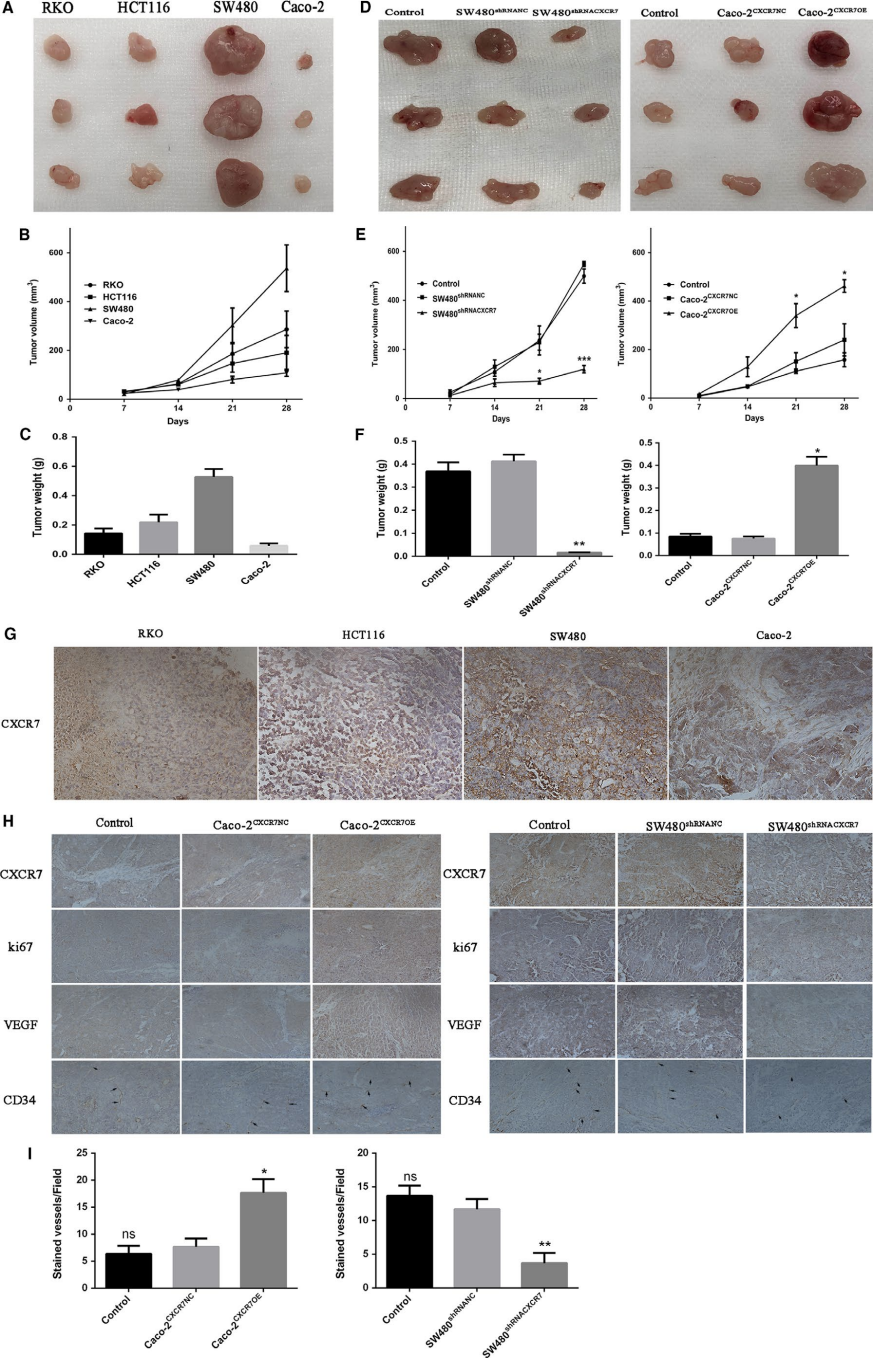
Effect of CXCR7 on tumor growth and angiogenesis in vivo Notes: A, Picture of RKO, HCT116, SW480 and Caco‐2 tumor tissue. B, Tumor volume of RKO, HCT116, SW480 and Caco‐2 groups. C, Tumor weight of RKO, HCT116, SW480 and Caco‐2 groups after sacrifice in mice. D, Left panel: Tumor picture of SW480 cells silencing CXCR7, right panel: Tumor picture of Caco‐2 cells overexpressing CXCR7. E, Left panel: Tumor volume of SW480 cells silencing CXCR7, right panel: tumor volume of Caco‐2 cells overexpressing CXCR7. F, Left panel: Tumor weight of SW480 cells silencing CXCR7 groups after sacrifice in mice, right panel: Tumor weight of Caco‐2 cells overexpressing CXCR7 after sacrifice in mice. G, Immunohistochemical analysis of CXCR7 in RKO, HCT116, SW480 and Caco‐2 tumor tissues. H, Tumor tissue sections were harvested for immunohistochemical staining of CXCR7, Ki67, VEGF and CD34 in each group. I. The count of CD34 microvessels reflected the MVD values. ^*^ *P* < .05, ^**^ *P* < .01 and ^***^ *P* < .001 vs overexpression groups. magnification 200×. Data in each group are presented as mean ± SD from three independent experiments. Abbreviations: VEGF: vascular endothelial growth factor; MVD: microvessel density NC, negative control. ns: No statistical significance

We initially implanted four colon cancer cell lines under the skin of nude mice, observing the tumor growth in each group of mice. The size of the tumors was measured every 7 days using an electronic digital caliper. Among the four colon cancer cell lines, SW480 cells produced the largest tumor, whereas Caco‐2 cells produced the smallest tumors (Figure 6A). The size and weight of tumors exhibited similar trends (Figure 6B and 6C). This result was positively correlated with the expression of CXCR7. In addition, we subcutaneously implanted in nude mice two stable cell lines (ie, Caco‐2 and SW480) that overexpressed or silenced CXCR7. Silencing of CXCR7 expression in SW480 cells resulted in the formation of smaller tumors than NC cells. In contrast, the overexpression of CXCR7 in Caco‐2 cells formed larger tumors (Figure 6D). In addition, silencing of CXCR7 in SW480 cells decreased the size and weight of tumors. In contrast, the overexpression of CXCR7 in Caco‐2 cells increased the size and weight of tumors. Notably, the differences between the control and NC groups were not statistically significant (Figure 6E and 6F). Immunohistochemical staining of tissues obtained from tumors confirmed the expression of CXCR7 in the four types of cells examined in this study (Figure 6G). To explore whether CXCR7 can affect the angiogenesis and proliferation of tumors, tissue sections were stained with antibodies against Ki67, VEGF, and CD34. The staining was higher in CXCR7‐overexpressing Caco‐2 cells vs the NC group. In contrast, the staining was lower in the CXCR7‐silenced SW480 cells vs the NC group (Figure 6H and 6I). Therefore, CXCR7 is a key factor in tumorigenesis by promoting cell proliferation and angiogenesis.

### 3.7 Angiogenesis induced by CXCR7 is associated with the activation of the AKT and ERK pathways in vivo and in vitro

**Figure 7:**
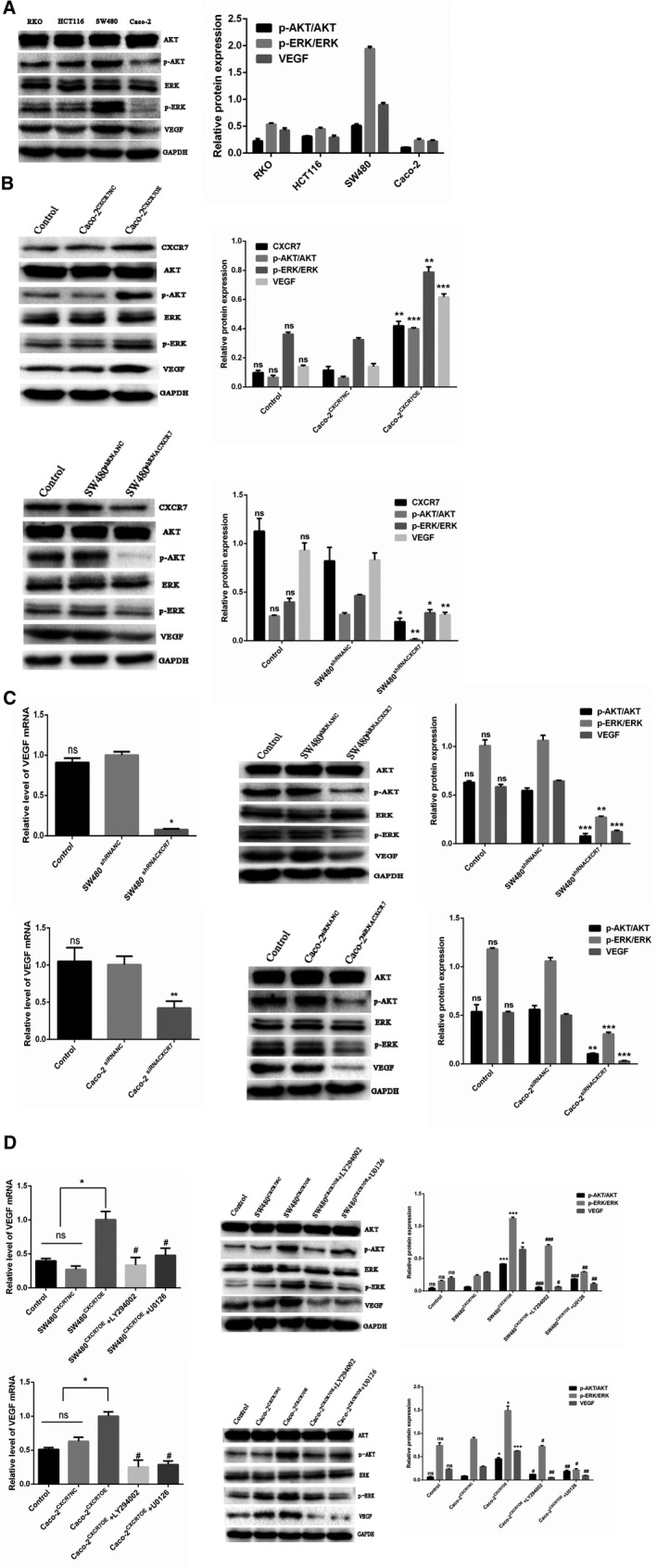
CXCR7 activates the AKT and ERK pathways to mediate angiogenesis Notes: A, Expression of total ERK, p‐ERK, total AKT, p‐AKT, VEGF, and GAPDH in RKO, HCT116, SW480, and Caco‐2 tumor tissues. B, Expression of total ERK, p‐ERK, total AKT, p‐AKT, VEGF, and GAPDH in CXCR7‐overexpressing or ‐silencing tissues. C, The expression levels of VEGF mRNA and total ERK, p‐ERK, total AKT, p‐AKT, VEGF, and GAPDH protein expression were significantly reduced in CXCR7‐silencing SW480 and Caco‐2 cells. D, The expression levels of VEGF mRNA and total ERK, p‐ERK, total AKT, p‐AKT, VEGF, and GAPDH protein expression were significantly increased in CXCR7‐overexpressing SW480 and Caco‐2 cells. Treatment with the inhibitors LY294002 and U0126 inhibited the mRNA expression of VEGF and protein expression of total ERK, p‐ERK, total AKT, p‐AKT, VEGF, and GAPDH. ^*^ *P* < .05, ^**^ *P* < .01, and ^***^ *P* < .001 vs the respective NC group. ^#^ *P* < .05, ^##^ *P* < .01, and ^###^ *P* < .001 vs the respective overexpression group. Abbreviations: VEGF, vascular endothelial growth factor; NC, negative control; ns, no statistical significance; CXCR7, chemokine receptor 7; CXCR7OE, overexpression of CXCR7

To analyze the mechanism of CXCR7‐mediated angiogenesis in colon cancer cells, the ERK/AKT and VEGF signaling pathways were analyzed. Firstly, in vivo, we detected the expression of various factors in four colon cancer tissues through western blotting. The results demonstrated that the expression of VEGF, p‐AKT, and p‐ERK was altered and positively correlated with the expression of CXCR7 (Figure 7A). In addition, we further examined the expression of various factors in the upregulated and downregulated groups. The western blotting results of this study showed that the Caco‐2^CXCR7OE^ group exhibited significant phosphorylation of AKT and ERK and increased expression of VEGF compared with the NC mice. In contrast, the SW480^shRNACXCR7^ group exhibited reduced phosphorylation of AKT and ERK and decreased expression of VEGF (Figure 7B). Secondly, in vitro, qRT‐PCR and western blotting showed that the knockdown of CXCR7 in SW480 and Caco‐2 cells decreased the phosphorylation of AKT and ERK and expression of VEGF (Figure 7C). In contrast, overexpression of CXCR7 in SW480 and Caco‐2 cells increased the phosphorylation of AKT and ERK and expression of VEGF (Figure 7D). Interestingly, in CXCR7‐expressing and ‐silent colon cancer cells, we noted that the AKT pathway can be activated using the U0126 inhibitor targeting the ERK pathway. However, the ERK pathway may also be activated using the LY294002 inhibitor targeting the AKT pathway. These results indicate that CXCR7 simultaneously regulates the ERK/AKT signaling pathways and the expression of VEGF in colon cancer in vitro and in vivo.

## 4 DISCUSSION

Colorectal cancer is the fourth leading cause of cancer death worldwide.^20^ The development of this type of cancer is a complex process, involving cell proliferation, invasion, angiogenesis, etc. In these processes, angiogenesis plays an important role in tumor invasion, growth, and migration.^21^ Angiogenesis is a complex process involving many steps, such as cell proliferation, migration, and lumen formation. The survival and proliferation of tumor cells are dependent on angiogenesis. Therefore, successful control of angiogenesis in cancer is a key step in the treatment of cancer.^22^ Numerous studies have shown that chemokines and their receptors are expressed in cancer and affect its development.^23^, ^24^ Recently, it has been reported that CXCR7 is overexpressed in many types of cancer (eg, ovarian, colorectal, and breast).^25^, ^26^, ^27^ Studies have shown that CXCR7—a G protein‐coupled receptor—has attracted attention due to its increased expression in colorectal cancer.^28^ The present study confirmed that CXCR7 is highly and variably expressed in colon cancer cells (ie, RKO, HCT116, SW480, and Caco‐2).

A growing body of evidence indicates that CXCR7 is involved in the process of angiogenesis in tumor. In addition, the stimulation of VEGF upregulates the expression of CXCR7 in endothelial cells.^29^ VEGF affects the germination and proliferation of endothelial cells, consequently stimulating tumor angiogenesis.^30^ In this experiment, the concentration of VEGF in the silencing group was significantly decreased, whereas that measured in the overexpression group was significantly increased. In addition, we found that the concentration of VEGF in the experimental group treated with the inhibitors LY294002 and U0126 was significantly decreased. Notably, VEGF protein expression analysis corroborated this finding.

CD34 is one of the important markers of angiogenesis, 31 while Ki67 is an important marker of tumor proliferation. Through an immunohistochemical analysis, we found that the staining of Ki67, VEGF, and CD34 was increased in transplanted tumor tissues overexpressing CXCR7. In contrast, the staining of Ki67, VEGF, and CD34 was reduced in the CXCR7‐silencing group.

Studies investigating breast cancer have found that the CXCL12/CXCR7 axis can serve as an intermediate link in the activation of endothelial cells. Moreover, inhibition of this axis inhibits tumor growth and angiogenesis.^32^ Experimental studies showed that animals treated with anti‐SDF‐1 showed higher expression of CXCR7 vs controls, indicating that CXCR7 regulates colon cancer angiogenesis and tumor growth independently of SDF‐1.^18^ In this study, we investigated angiogenesis through CXCR7 in colon cancer. We selected two cell lines (ie, Caco‐2 and SW480) with the lowest and highest expression of CXCR7, respectively. Both types of cells were treated to overexpress or silence CXCR7, and the stable cells were co‐cultured with HUVEC. The results showed that CXCR7‐silenced Caco‐2 and SW480 cells inhibited proliferation, migration, and tube formation in HUVEC. In contrast, CXCR7‐overexpressing Caco‐2 and SW480 cells promoted the proliferation, migration, and tube formation in HUVEC. In addition, these abilities of endothelial cells treated with the inhibitors LY294002 and U0126 showed a significant downward trend than those observed in the overexpression group. These findings indicate that CXCR7 is involved in tumor angiogenesis and may be mediated by the AKT and ERK pathways. Our experiments showed that the inhibitors LY294002 and U0126 inhibit the process of angiogenesis and expression of VEGF. Therefore, we hypothesize that CXCR7 regulates the expression of VEGF through the AKT and ERK pathways, thereby regulating the growth and angiogenesis in colon cancer.

In SW480 cells overexpressing CXCR7, we found that phosphorylation of AKT and ERK was significantly increased. Notably, the degree of phosphorylation was decreased after treatment with the respective inhibitors. In contrast, in CXCR7‐silenced SW480 cells, AKT and ERK phosphorylation was significantly reduced. The same trend was observed in the overexpressing and silencing groups of Caco‐2 cells. These results show that the expression of CXCR7 regulates angiogenesis in colon cancer through the AKT and ERK pathways. The present findings are consistent with those reported by Hao et al.^14^ In J82 cells expressing CXCR7, they indicated that the AKT pathway may be activated through the use of the inhibitor U0126. However, the ERK pathway can also be activated through the use of the LY294002 inhibitor. The results indicated that the AKT and ERK pathways of CXCR7 in bladder cancer cells can be mutually regulated. However, a study reported contradictory findings related to the migration process. Romain et al found that silencing of CXCR7 did not alter the migration process or activate the AKT or ERK pathway.^33^Targeting CXCR7 and CXCL12/CXCR7 interactions did not affect AKT or ERK signaling. Furthermore, in partial agreement with our findings, studies reported that expression of CXCR7 and co‐expression with CXCR4 in breast cells can regulate the phosphorylation of AKT but not that of ERK.^34^ The development of cancer is a complex process, closely related to variable factors and characteristics between different types of this disease. Further investigation of the role of the AKT and ERK pathways in different cell lines is warranted.

In conclusion, the present research shows that CXCR7 induces angiogenesis in colon cancer through the activation of the AKT and ERK pathways. These data may provide a new theoretical basis for the mechanism of angiogenesis in colon cancer. Moreover, CXCR7 may be a new therapeutic target in this setting.

## CONFLICT OF INTEREST

The authors declare that the research was conducted in the absence of any commercial or financial relationships that could be construed as a potential conflict of interest.

## AUTHOR CONTRIBUTIONS

Xiang Li: Conception and design of the study, and data analysis. Xuemei Wang: Drafting the article and revising it critically for important intellectual content, and final approval of the version to be submitted. Zitao Li: Acquisition, analysis, and interpretation of data. Zhen Zhang: Contributed reagents/materials. Yixia Zhang: Data curation.
