# Supplementary figures and images for: Chemokine receptor 7 targets the vascular endothelial growth factor via the AKT/ERK pathway to regulate angiogenesis in colon cancer
Source: Cancer Med. 2019 Jul 26;8(11):5327–40. doi: 10.1002/cam4.2426 (PMC6718596; doi:10.1002/cam4.2426)

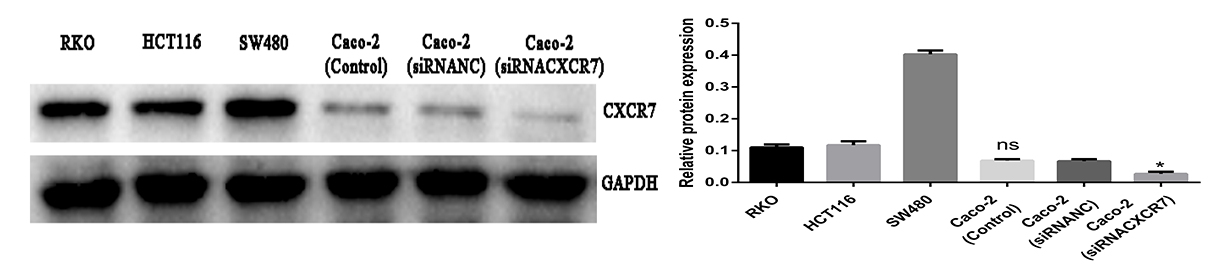

Supplement: Supplementary file 1 [file CAM4-8-5327-s001.tif]

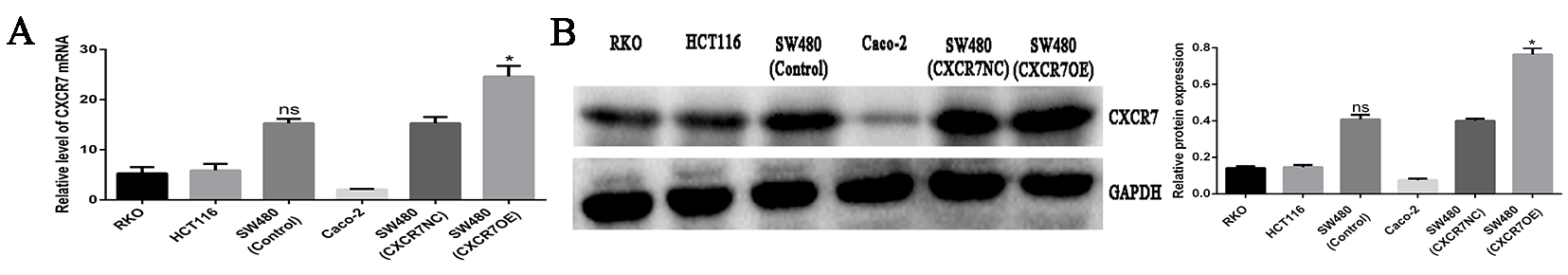

Supplement: Supplementary file 2 [file CAM4-8-5327-s002.tif]
